# Excitation-inhibition imbalance in the motor control network: a key factor in post-stroke spasticity

**DOI:** 10.3389/fnhum.2025.1615483

**Published:** 2025-08-13

**Authors:** Shanshan Zeng, Lu Li, Lingying Wu, Ran Li, Xukun Tang, Xiongxing Sun, Shigao Lin, Zhuxuan Liu, Jie Tang, Qian Liu, Dahua Wu, Le Xie

**Affiliations:** Hunan Provincial Hospital of Integrated Traditional Chinese and Western Medicine (The Affiliated Hospital of Hunan Academy of Traditional Chinese Medicine), Changsha, Hunan, China

**Keywords:** spasticity, stroke, stretch reflex hyperactivity, excitation-inhibition imbalance, neural excitability

## Abstract

Spasticity is a recognized motor dysfunction that frequently arises following a stroke and significantly impacts the quality of life of affected patients. It is characterized by involuntary muscle activation resulting from overexcitation of the stretch reflex. Currently, therapeutic options for post-stroke spasticity are limited, and the underlying pathological mechanisms remain inadequately understood. Evidence suggests that stretch reflex hyperactivity is attributable to a combination of factors, including abnormal supraspinal projections, imbalances within the intraspinal network, and peripheral muscle alterations. This review aimed to examine supraspinal projections, specifically addressing the imbalance between excitatory and inhibitory output signals within the neural network of the brain’s motor control region, and to discuss the significant role of the associated nerve bundles in the disease’s pathogenesis.

## 1 Introduction

Post-stroke spasticity (PSS) is a motor and sensory disorder characterized by a velocity-dependent increase in the muscle response to stretch, accompanied by exaggerated tendon reflexes resulting from hyperexcitability of the stretch reflex. It represents a significant complication of stroke, affecting approximately 17.0%–42.6% of patients with stroke, and can be a major contributor to disability (Bavikatte et al., 2021; Francisco and McGuire, 2012; Rakers et al., 2023; Urban et al., 2010). The spasticity resulting from cerebral infarction presents a highly intricate clinical challenge. Beyond movement disorders, including spasticity, contracture, dystonia, and abnormal reflex responses, individuals with cerebral infarction may exhibit a range of neurological deficits, including sensory, visual, cognitive, mental, and behavioral impairments (Francisco et al., 2021a,b). These symptoms arise from the disruption of neural networks, which can affect the adjacent non-infarcted regions. Spasticity lacks a standardized definition, and the underlying pathophysiological mechanisms remain unclear. However, hyperexcitability of the stretch reflex, resulting from an abnormality in the output signal of excitatory and inhibitory pathways in the brain, remains a fundamental connotation of the definition and neuropathological mechanism of PSS (Thibaut et al., 2013).

## 2 Definition of PSS

Research on the definition of PSS has yielded inconclusive results so far (Balakrishnan and Ward, 2013). Lance defined spasticity as a movement disorder characterized by a velocity-dependent increase in the tension stretch reflex (muscle tone) accompanied by pronounced tendon twitching caused by muscle tone overexcitability (Lance, 1980). Nevertheless, as research evidence progresses and updates, scholars assert that apart from muscle speed, muscle length is also a significant determinant of spasm occurrence. For instance, when a muscle is shorter, a greater number of spasms occur in the quadriceps muscle. However, in the flexor muscles of the upper limbs (biceps brachii) and ankle extensors (gastrocnemius and soleus muscles), a higher incidence of spasms occurs when the muscle is longer (Ganguly et al., 2021). Furthermore, Lance also overlooked the controlling role of “sensation” in his definition (Bhimani and Anderson, 2014). Therefore, in 2005, British scholar Pandyan et al. (2005) proposed the following definition: “A disorder of sensory-motor control caused by upper motor neuron dysfunction, characterized by intermittent or sustained involuntary muscle activation.” This definition considers the role of sensation in movement disorders and supplements the idea that spasticity is not merely a phenomenon of increased joint and muscle tone or difficulty in movement. Additionally, it describes the clinical manifestations of intermittent or continuous muscle hyperactivity rather than simply attributing the underlying mechanism to excessive muscle tone excitability (Ganguly et al., 2021). In 2018, the IAB (International Advisory Board)–Interdisciplinary Movement Disorder Working Group proposed a new consensus on the definition of spasticity: “It is defined as involuntary muscle hyperactivity in the presence of central paresis” and highlighted that involuntary muscle hyperactivity may encompass conditions including *spasticity* sensu strictu, rigidity, dystonia, and spasms, or a combination thereof (Dressler et al., 2018). To elucidate the advancement in defining spasticity and to distinguish PSS from other neuromuscular injuries, a novel definition was proposed by American researcher Li et al. (2021): spasticity is characterized by an increase in muscle velocity and resistance to externally applied muscle stretching, contingent upon muscle length. This phenomenon is caused by hyperexcitable descending excitatory brainstem pathways and the resultant exaggerated stretch reflex response (Li et al., 2021). This definition also indicates the significance of muscle length and speed in regulating muscle tone. It emphasizes the impact of hyperexcitation of the descending brainstem pathway on the development of PSS and the inseparable connection between spasticity and other movement disorders (Sheean and McGuire, 2009; Wissel and Hernandez Franco, 2024).

## 3 Abnormal excitation-inhibition of descending conduction pathway in PSS

The motor system primarily consists of the pyramidal system, lower motor neurons, extrapyramidal system, and cerebellum. These components must coordinate and collaborate to achieve various fine and complex movements (Herculano-Houzel et al., 2016; Rimmele et al., 2018). Additionally, all movements are generated after receiving sensory impulses (Jellinger, 2019). In the nervous system, the somatic motor conduction pathway denotes the neural connections between the cerebral cortex and effectors of the body (and effectors of visceral activities), mainly regulating fine movements through the pyramidal system and coordinating fine movements through the extrapyramidal system (de Oliveira-Souza, 2012). The pyramidal system comprises neuronal cell bodies and their axons located in the motor cortex of the precentral gyrus within the frontal lobe, encompassing both the corticospinal tract (CST) and corticobulbar tract (CBT) (Shepherd, 2014). The CST is a collection of nerve fibers that extends from the cerebral cortex to the anterior horn of the spinal cord. The fiber bundle that transmits signals from the cerebral cortex to regulate movement in brainstem nuclei is known as the cortical, nuclear bundle, cortical brainstem bundle, or cortical bulbar bundle. The pyramidal system comprises pyramidal cells and their axons located in the motor region of the central prefrontal gyrus, encompassing the CST connecting the cerebral cortex to the anterior horn of the spinal cord and the CBT linking the cerebral cortex to the motor nucleus of the brainstem (Paul et al., 2023). Patients with upper motor neuron (UMN) syndrome often experience abnormal patterns of muscle activity, clinically manifested as negative (reduced muscle activity) and positive (excessive muscle activity) signs (Crowe et al., 2023). Spasticity is a representative sign of excessive muscle activity in UMN syndrome, while other signs of excessive muscle activity include the Babinski sign, myoclonus, dystonia, hyperreflexia, and athetosis (Esquenazi et al., 2009; Segal, 2018; Wissel and Hernandez Franco, 2024).

**FIGURE 1:**
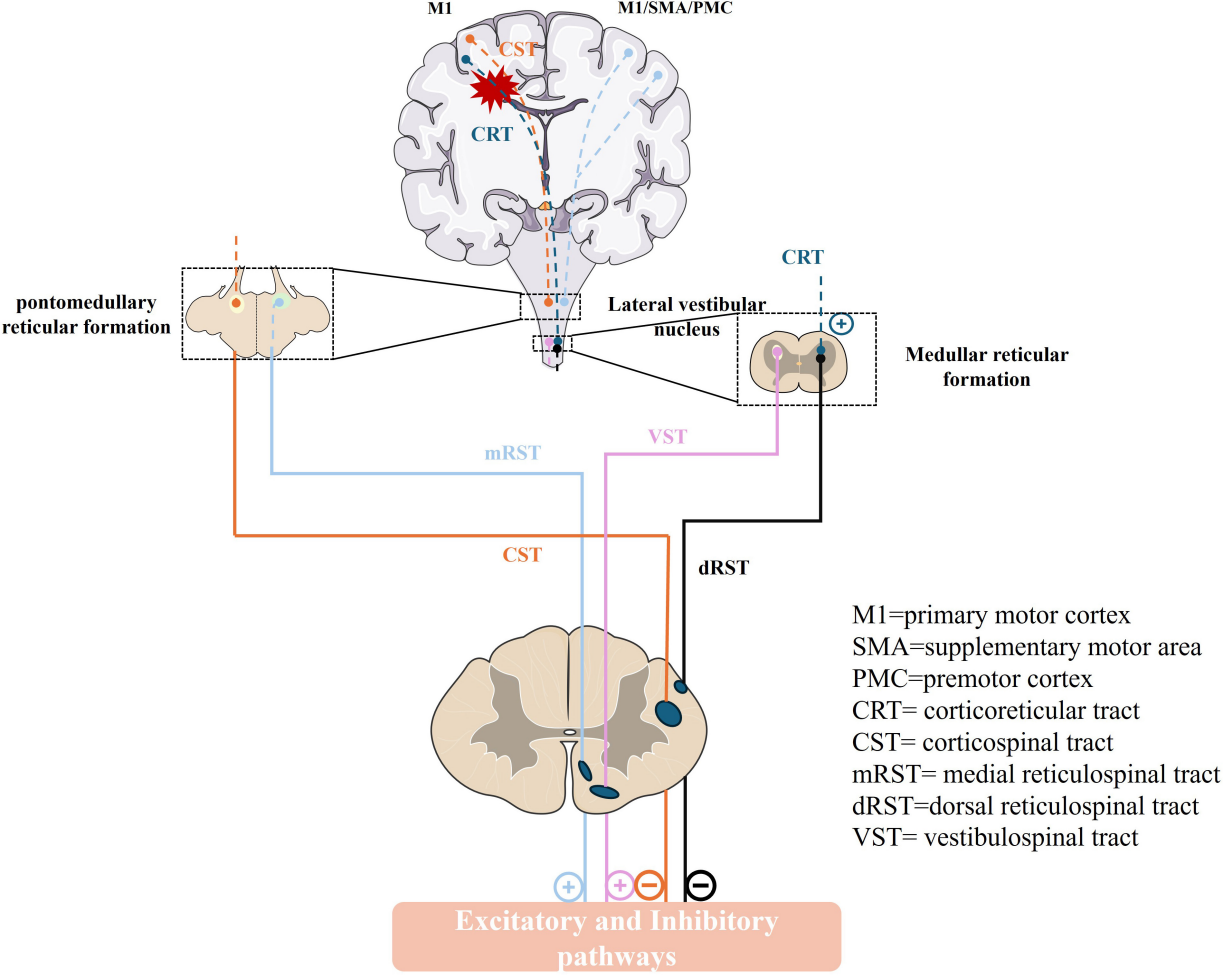
The descending pathway that regulates the human stretch reflex circuit and muscle tone. M1, primary motor cortex; SMA, supplementary motor area; PMC, premotor cortex; CRT, corticoreticular tract; CST, corticospinal tract; mRST, medial reticulospinal tract; dRST, dorsal reticulospinal tract; VST, vestibulospinal tract. (+): excitatory; (–): inhibitory.

Descending systems from the brain exert a major influence over sensory and motor processes within the spinal cord. The stretch reflex in the human body is normally influenced by the interplay of excitation-inhibition drives, including two inhibitive drives CST from the motor cortex, and the dorsal reticulospinal tract (dorsal RST) from the medullary reticular formation (Sangari and Perez, 2019), and three facilitatory drives, primarily from the corticoreticular tract (CRT) from the premotor cortex (PMC), the medial reticulospinal tract (medial RST) of the pontine reticular formation and vestibulospinal tract (VST) from the lateral vestibular nucleus (Deiters’ nucleus). In these pathways, the dorsal RST, medial RST, and VST are pivotal, whereas the CST exerts a minimal impact on the excitability of the stretch reflex (Ganguly et al., 2021; Mukherjee and Chakravarty, 2010). The dorsal RST, originating from the ventromedial medullary reticular, receives projections from the PMC and supplementary motor area (SMA) through CRT. It descends parallel to the dorsolateral CST and exerts a potent inhibitory effect on the stretch reflex (Jang and Lee, 2019; Ko et al., 2021). In contrast, the medial RST originates in the pontomesencephalic tegmentum, receives inputs primarily from the ipsilateral PMC/SMA, and descends along the VST in the medial ventral cord, exerting excitatory effects (Ko et al., 2021; Li and Francisco, 2015; Figure 1). Owing to their close anatomical proximity, the CRT and CST are often simultaneously damaged in stroke, leading to a loss of cortical control over the medullary inhibitory center and a weakened inhibitory effect of the dorsal RST. The facilitatory medial RST and VST are not under cortical control, resulting in unopposed excitatory effects and exaggerated stretch reflexes. At this point, the excitatory action of the medial RST plays a crucial function (Li and Francisco, 2015; Li, 2017).

## 3.1 Anatomy and inhibitory function of CRT

Currently, research on the anatomical structure of the CRT is limited. Yeo et al. (2012) conducted a study in 2012 using diffusion tensor tractography (DTT) to generate a three-dimensional reconstruction of the brains of 24 healthy individuals to identify CRT. This study focused on the medullary reticular formation as the region of interest (ROI), with the first target ROI being the midbrain tegmentum and the second target ROI being Brodmann area 6 in the PMC. After measuring the fractional anisotropy (FA) and mean diffusivity (MD), it was observed that CRT may originate from the PMC, descend through the corona radiata and the anterior part of the internal capsule before the CST (with a distance interval of 6–12 mm in front and behind the CST), reach the midbrain tegmentum, and terminate in the reticular formation of the brainstem (Yeo et al., 2012). Other DTT studies on the anatomical structure of CRT have demonstrated that the average tract volume (TV) values of the cortical origin areas of CRT were as follows: PMC (1177.3), primary motor cortex (M1; 994.9), primary somatosensory cortex (S1; 580.8), and prefrontal cortex (PFC; 575.7). The TV value of the PMC area was significantly higher than that of other cortical areas, and the TV of M1 was significantly higher than that of S1 and PFC. However, there was no significant difference in TV values between S1 and PFC (Jang and Seo, 2014; Neil, 2008). These studies have verified that PMC is the main source of CRT, along with other potential sources, including M1, S1, and PFC (Boyne et al., 2022; Jang and Lee, 2019; Jang and Seo, 2014). Research involving animal models has demonstrated that CRT predominantly originates from the primary and secondary motor cortices, the anterior cingulate gyrus, the primary somatosensory cortices, and the medial prefrontal cortices. The projection of CRT is notably stronger in these regions and exhibits a more pronounced bilateral presence compared to the CST (Boyne et al., 2021). These attributes imply that CRT may be integral to motor recovery following central nervous system injury.

The CRT predominantly contributes to approximately 30%–40% of the motor activity in muscles proximal to the joint. Furthermore, CRT plays a significant role in gait function and postural stability (Jang and Lee, 2019). Jang et al. (2015) investigated 17 patients exhibiting complete CST but presenting with motor weakness among 134 cases of subarachnoid hemorrhage to examine the correlation between motor weakness and diffusion tensor imaging (DTI) parameters. They discovered that FA values of white matter microstructural directionality and integrity were associated with shoulder and hip motor function in relation to CRT. However, no correlation was observed between the FA values and motor function of the distal joint muscles (elbow, hand, knee, and ankle). Consequently, the study concluded that the severity of CRT injury in these patients is related to weakness of the proximal joint muscles (shoulder and hip joints). Although reticulospinal projections are thought to primarily regulate the proximal limb muscles, recent studies have demonstrated some control over the distal muscles, including the wrist and intrinsic hand muscles. Enhanced corticoreticular projections have been revealed to potentially compensate for damage to the sensorimotor cortex. Darling et al. (2018) conducted motor tests on rhesus monkeys with frontal and frontoparietal lesions and utilized neural tracing techniques to demonstrate that motor recovery of arm and hand function following sensorimotor cortex injury was significantly associated with an increased intensity of descending projections from the uninjured medial cortical motor area to the brainstem reticular nucleus. These findings indicate that enhanced corticoreticular projections are crucial for the restoration of distal muscle control. Nevertheless, accurately estimating the motor function associated with CRT poses significant challenges, as patients with brain injuries frequently exhibit concomitant damage to other neural tracts, including exocortical fibers or the CST originating from secondary motor areas (Jang, 2014; Newton et al., 2006). Although the status of CRT has been found to be more closely correlated with the severity of spasticity (Cho et al., 2023), other neural pathways, such as CST, also significantly contribute to motor function impairment.

### 3.2 Anatomy and inhibitory function of CST

The CST serves as the principal nerve bundle within the human brain that governs autonomic motor functions and orchestrates primary motor activities from the cervical region to the distal extremities, with a particular emphasis on the precise control of fine distal movements (Betti et al., 2022; Saltão da Silva et al., 2022). Most axonal fibers of the CST arise from M1, while some originate from the SMA, PMC, and cingulate motor regions (Jang, 2014; Van Wittenberghe and Peterson, 2022). The somatic structures of these pyramidal neurons are situated within cortical layer V, with axonal projections traversing in bundles through the internal capsule, cerebral pedunculus, and ventral pons, ultimately terminating in the ventral medulla. Most fibers decussate at the junction between the brain stem and the spinal cord, contributing to the formation of the lateral CST. This decussation enables bilateral coordination, allowing each hemisphere of the brain to exert control over the contralateral side of the spinal cord, and a minority (approximately 5%–15%) of uncrossed fibers persists on the ipsilateral side, forming the anterior CST. The axonal fibers of the anterior and lateral CST penetrate the gray matter of the anterior horn of the spinal cord, where they establish synaptic connections with lower motor neurons (Jankowska and Edgley, 2006). These lower motor neurons subsequently project from the spinal cord to regulate the trunk muscle contractions (Welniarz et al., 2017). The PMC and cingulate motor areas are involved in cognitive-related motor control processing and motor decisions. Moreover, SMA is hypothesized to significantly influence the temporal regulation of muscle activation, particularly during stretching workouts (Boccuni et al., 2019; Strick et al., 2021). The anterior CST is involved in motor regulation of the axial muscles, whereas the lateral CST serves as the principal pathway for transmitting motor information to the limbs (Van Wittenberghe and Peterson, 2022). Numerous studies have established a correlation between CST integrity and motor function recovery after stroke (Paul et al., 2023). In the acute and early subacute phases of stroke, damage to the CST leads to neuronal cell death, axonal injury, and demyelination of corticospinal neurons. Pyramidal cells are located in the ipsilateral primary motor cortex (M1), thereby disrupting neuronal information transmission (Liu et al., 2021). CST microstructures were identified to predict improvements in distal upper limb movement among chronic stroke survivors. Those with suboptimal ipsilateral CST microstructures at baseline, characterized by reduced symmetry in CST FA, exhibited greater movement enhancements with minimal practice than individuals possessing more favorable CST microstructural integrity (Kim et al., 2021). A study based on DTI and Least Absolute Shrinkage and Selection Operator (LASSO) regression multimodal predictive models demonstrated that CST microstructures predict distal upper limb movement improvement in chronic stroke survivors (Kim et al., 2021). Stroke-related CST injuries frequently impair hand and upper limb functions, particularly hindering fine motor control recovery (Carmichael, 2016; Ito et al., 2022; Lam et al., 2018). Actions, including “reaching and grasping,” depend on complex sensorimotor information and visual-motor coordination. CST fibers are crucial for translating an object’s intrinsic properties, including size, into hand and finger movements. Recent research indicates that involvement of the CST alone is insufficient to induce spasticity. The etiology of spasticity following cortical injury from infarction may involve the combined participation of cortical reticular fibers, which connect the PMC and bulbar reticular formation, from which dorsal RST originates (Ganguly et al., 2021). Hypotonia, weakness, and loss of superficial reflexes exclusively result from CST involvement. If the dorsal RST is also affected, it leads to a loss of inhibition and non-antagonistic medial RST activity, causing spasticity and hyperreflexia (Ganguly et al., 2021).

### 3.3 Anatomical structure of reticulospinal tract and its facilitation role

Reticulospinal tract (RST), another critical descending system alongside the CST, influences movement and posture by activating the proximal and distal muscles of both upper limbs (Akalu et al., 2023; Glover and Baker, 2020; Glover and Baker, 2022). The RST consists of two descending systems: The dorsal RST, originating from the dorsolateral reticular structure of the medulla and receiving motor cortex projections, and the medial RST, originating from the pontine tegmentum and connected to the pontomedullary reticular formation, it receives inputs primarily from ipsilateral PMC and SMA, and descends ipsilaterally (Glover and Baker, 2022). The dorsal RST, along with the lateral CST and corticoreticulospinal tract, descends in the dorsolateral spinal cord, whereas the medial RST descends with the VST in the ventromedial spinal cord (Brownstone and Chopek, 2018). The RST is predominantly localized at the synapses of interneurons within the spinal cord axis and proximal musculature, with some fibers exhibiting direct connections with motor neurons (MN). The dorsal RST exerts a significant inhibitory effect. In contrast, the medial RST and VST provide excitatory inputs to the intraspinal network, functioning as supraspinal excitatory systems. Therefore, the medial and lateral RST offers a balance of excitatory and inhibitory inputs to the spinal MN network. In cases of stroke involving cortical injury, both CST and CRT are frequently compromised due to their anatomical proximity, leading to varying degrees of motor function impairment. This impairment results in the loss of antagonistic inhibition of the facilitative medial RST and VST pathways and thereby become highly excitable (Li and Francisco, 2015; Li et al., 2019). While compensatory excitation of the RST can partially substitute for the CST and aid in the restoration of motor function post-stroke (Choudhury et al., 2019; Ellis et al., 2012; Ellis et al., 2018; McPherson et al., 2018; Owen et al., 2017), excessive excitation may induce spasticity, abnormal motor coordination, and motor control disorders. This underscores the critical role of the RST in the development of spasticity (Chen et al., 2018).

A high-resolution structural Magnetic Resonance Imaging (MRI) and unbiased whole brain voxel analysis of the brainstem and cervical spinal cord (C1–C8) revealed that dyskinesia in patients with stroke correlates with the white matter integrity of the affected corticospinal and bulbar spinal tracts, particularly the medial and lateral RST and the descending medial VST. Lower white matter integrity in the lateral RST and higher integrity in the contralateral medial RST correlated with more severe motor, joint, sensory, and balance disorders in the upper limbs (Karbasforoushan et al., 2019). In addition to DTI, the acoustic startle reflex (ASR) is a widely used technique for assessing the excitability of the RST following a stroke (Chen et al., 2019a; Chen et al., 2019b; Li et al., 2014). The ASR represents an involuntary motor response elicited by sudden, intense acoustic stimuli (SAS), a phenomenon also referred to as the “StartReact effect” (Nonnekes et al., 2014; Tapia et al., 2022). This method is frequently employed to investigate alterations in cognitive and psychological behaviors, including learning, memory, emotion, and sensation (GhotbiRavandi et al., 2021; Gómez-Nieto et al., 2020). Current research suggests that the human ASR circuit involves the cochlear nucleus, caudal pontine reticular nucleus, brainstem MN, and spinal cord pathways, which the medial RST activates (Li et al., 2019). The motor recovery process following a stroke can be broadly categorized into three primary stages, which are further subdivided into six stages as delineated by Brunnstrom: the flaccid stage (Stage I), the spasticity stage (Stages II–IV), and the chronic recovery stage (Stages V–VI) (Brunnstrom, 1966). In Stage I, the ASR is normal, suggesting the integrity of the reticulospinal pathway. The heightened ASR observed in Stages II–IV indicates increased excitability of the RST. In Stages V–VI, the ASR decreases, reflecting a reduction in compensatory excitation of the RST and a recovery of CSTmotor function (Li, 2017; Zhang et al., 2022).

Exaggerated ASR responses, including increased frequency, amplitude, and duration, were observed in the spastic muscles of patients with PSS (Jankelowitz and Colebatch, 2004; Li et al., 2014; Sohtaoğlu et al., 2016). Li et al. (2014) investigated the excitability of the RST in patients with chronic stroke across various stages of motor recovery, including soft fistula, spasticity, and chronic recovery stages. Their findings demonstrated that the ASR response remained within normal parameters in stroke survivors without muscle spasms, irrespective of the relaxation or recovery stages. Conversely, a bilateral exaggerated ASR response was frequently observed in patients experiencing spasms. This exaggerated response manifested earlier and persisted longer on the affected than on the unaffected side. Chen et al. (2019a) conducted a study utilizing SAS on the right motor cortex of healthy individuals and the contralateral primary motor cortex (cM1) in individuals with stroke. During a 10% maximum voluntary contraction task involving the elbow flexor in a resting state, a 50 ms transcranial magnetic stimulation was administered to the left motor cortex or the unaffected side. The findings indicated that the excitability of the RST in stroke patients, along with its interactions with cM1 and the descending corticospinal system, were comparable to those observed in healthy individuals. This revealed that the contralateral RST excitability in patients with stroke was possibly within the normal range. The results demonstrated that RST hyperexcitability in patients with stroke was present during the spasticity stage rather than during the fistula soft stage or chronic recovery stage. Furthermore, the data imply that RST projections may predominantly exhibit unilateral characteristics.

### 3.4 Anatomical structure of VST and its facilitation role

The VST is integral to the processing of visual information and the determination of necessary movements to maintain and control balance in an upright posture (Yeo et al., 2020). It serves as the extrapyramidal motor pathway responsible for the regulation of gait and balance (Nakamura et al., 2021; Omura et al., 2022; Tanaka et al., 2021). The vestibulocochlear nerve conveys information regarding head direction changes to the vestibular nucleus, which subsequently transmits motor instructions through the VST to maintain equilibrium between the body and the head’s upright position. The VST comprises two distinct descending projection systems. In a study by Jang et al. (2018) 40 healthy volunteers were recruited to reconstruct the medial and lateral components of the VST in the human brain by using DTT. Their observations revealed that the medial VST originated from the medial vestibular nucleus, traversed the posterior medial medulla, and terminated at the anterior cord of the cervical spinal cord (C1–C8). In contrast, the lateral VST originated from the lateral vestibular nucleus, passed through the posterolateral medulla, and terminated at the anterior aspect of the lateral cord of the spinal cord (McCall et al., 2017). The FA value of the medial VST was significantly higher than that of the lateral VST, whereas MD and TV values were substantially lower than those of the lateral VST. Projection of the medial VST terminated in the cervical spinal cord (C1–C8) and upper thoracic spinal cord, suggesting its primary role in axial and upper limb motor control (Cleland and Madhavan, 2021).

Nearly all VST axons (97%) and a significant proportion of RST axons (59%) possess excitatory neurotransmitter transporters, whereas a relatively small percentage of RST axons (20%) contain inhibitory transporters (Du Beau et al., 2012). In a comprehensive neural pathway, RST and VST mainly facilitate the descending modulation of excitatory and inhibitory balances. The dorsal RST descends parallel to the lateral CST and exerts a predominantly inhibitory influence on the spinal stretch reflex. Conversely, the medial RST and VST descend within the ventromedial spinal cord, thereby providing excitatory inputs. The dorsal RST receives projections from the motor cortex through the corticoreticular pathway, which is intricately linked to the CST. This connection is frequently compromised during stroke, leading to a diminished cortical facilitation effect on the inhibitory centers of the medulla oblongata, and the inhibition of the dorsal RST is decreased. Consequently, as the facilitated medial RST and VST operate independently of cortical regulation, the excitatory pathway experiences a reduction in antagonistic influence, resulting in the excitation of the stretch reflex (Li and Francisco, 2015).

The current study proposes that heightened MN excitability may constitute the central mechanism underlying spasticity following cerebral infarction (Hu et al., 2015; Zhang et al., 2023). This increased excitability is primarily attributed to persistent depolarization of the MN membrane potential, which innervates the spasmodic nerve and approaches its discharge threshold (Afzal et al., 2019; Mottram et al., 2009). Alternatively, a reduction in synaptic input from muscle afferents, necessary to achieve the MN firing threshold in the spastic muscle, may also contribute to this phenomenon (Afzal et al., 2019). Mottram et al. (2009) demonstrated that alterations in MN discharge could result from the influence of low-level depolarization synapses in the resting MN pool This finding implies that therapeutic strategies aimed at diminishing tonic synaptic depolarization or reducing the resting membrane potential of the MN may play a crucial role in preventing spasticity. Nevertheless, the specific neural pathway responsible for mediating this increase in MN excitability remains to be elucidated (Afzal et al., 2019). A recent study addressing the levels of vestibular-evoked myogenic potentials (VEMPs) in the neck muscles of patients with chronic stroke has contributed significantly to this field of research (Miller et al., 2014). This study identified asymmetry in the lateral and contralateral amplitudes of VEMPs in spastic muscles, possibly due to lateral damage to corticobulbar pathways. This damage potentially causes an imbalance in the descending vestibular inputs to the MN pool. Consequently, the resting membrane potential of the MN on the affected side is closer to the threshold for neuronal activation. Furthermore, the study corroborates the unilateral characteristics of the vestibular pathway, specifically highlighting that the lateral VST aligns with the distinctly lateralized nature of spasticity (Miller et al., 2014).

## 4 Conclusion

In summary, PSS pathogenesis is intricate and involves multiple neural networks, including the brain, spinal cord, and peripheral systems. Recent studies have elucidated inhibitory pathways, including CRT originating from the PMC, CST from the motor cortex, and dorsal RST from the dorsolateral medulla reticular formation. Additionally, facilitative mechanisms, including the interplay between the medial RST from the pontine reticular formation and the VST from the lateral vestibular nucleus or Deiters’ nucleus, are integral to PSS pathophysiology. Currently, some interventional studies targeting motor pathways, such as repetitive transcranial magnetic stimulation, have demonstrated promising therapeutic potential (Peng et al., 2024; Tam et al., 2024; Williamson et al., 2023). However, the specific mechanisms require further investigation.

Furthermore, in addition to the central nervous system, peripheral factors significantly contribute to the pathophysiology of PSS. For example, the sensitivity of the muscle spindle to stretching is indicative of the activity equilibrium between antagonistic muscles. An inability to appropriately activate the spindle, or its excessive activation, can lead to dysregulation of the spastic muscle stretch reflex (Dimitriou, 2014; Stecco et al., 2014). Additionally, factors such as changes in the extracellular matrix, increased muscle stiffness, and reduced movement flexibility due to collagen deposition are critical contributors to the development of PSS and associated movement disorders (Raghavan et al., 2016; Raghavan, 2018; Trompetto et al., 2014). It is essential to emphasize that postactivation depression represents a mechanism that reduces the release of Ia afferent neurotransmitters and has been shown to play a critical role in the development of spasticity. Importantly, this process is independent of the inhibitory spinal cord circuitry and is not modulated by descending motor pathways (Trompetto et al., 2014).

Recent evidence indicates that enhanced neuroplasticity, particularly through increased reconnection between cortical and spinal cord neurons, holds the potential to restore neurological functions. This understanding establishes a neuroanatomical basis and pathophysiological target for regulating neuroplasticity, thereby addressing spastic paralysis following cerebral infarction. Consequently, it is crucial to explore strategies that promote the recovery of spasticity by enhancing neuroplasticity, thereby facilitating a balance between excitation and inhibition within neural networks.
